# Novel Trimethoprim Resistance Gene *dfrA49* Identified in Riemerella anatipestifer from China

**DOI:** 10.1128/spectrum.04747-22

**Published:** 2023-03-14

**Authors:** Yongjia Jiang, Kai Peng, Qiaojun Wang, Mianzhi Wang, Ruichao Li, Zhiqiang Wang

**Affiliations:** a College of Veterinary Medicine, Yangzhou University, Yangzhou, People’s Republic of China; b Institute of Comparative Medicine, Yangzhou University, Yangzhou, People’s Republic of China; c Jiangsu Co-innovation Center for Prevention and Control of Important Animal Infectious Diseases and Zoonoses, Yangzhou University, Yangzhou, People’s Republic of China; d Joint International Research Laboratory of Agriculture and Agri-Product Safety of Ministry of Education of China, Yangzhou, People’s Republic of China; University of Guelph College of Biological Science

**Keywords:** plasmids, *Riemerella anatipestifer*, *dfrA49*, trimethoprim resistance

## Abstract

Resistance to trimethoprim is mainly mediated by the acquisition of mobile *dfrA* genes, and most of them were discovered in Enterobacteriales. A total of 139 Riemerella anatipestifer isolates were collected from different farms in China during 2014 to 2020. Whole genome sequencing (WGS) and genome analysis of R. anatipestifer isolates revealed a 504-bp open reading frame (ORF) encoding a putative *dfrA* gene. This DfrA variant shared 66.47% amino acid sequence identity with DfrA36 and shared ≤51.20% identity with any other previously identified DfrA proteins. The novel *dfrA* gene, designated *dfrA49*, conferred trimethoprim (TMP) resistance when cloned into Escherichia coli BL21(DE3). Thirty *dfrA49*-positive isolates were identified from Jiangsu and Guangdong province (5/38, 13.16%, and 25/101, 24.75%, respectively). Five of the 38 isolates had obtained the complete genome sequences. Genomic analysis showed that the *dfrA49* gene was located on chromosomes or a plasmid (four of them were on chromosomes and one was located on a plasmid). The plasmid p20190305E2-2_2 carried *dfrA49*, *catB*, *ermF*, *ereD*, *bla*_OXA_ (88.36% identity with *bla*_OXA-209_), Δ*arr*, and *tet*(X18). Further research indicated that *dfrA49* usually coexisted with *catB* in R. anatipestifer. In this study, a novel trimethoprim resistance gene, *dfrA49*, was identified and characterized in chromosome and plasmid sequences from R. anatipestifer using WGS and bioinformatic methods. It further expands knowledge about the pool of mobile *dfrA* genes that confer resistance to trimethoprim and provides information about antibiotic resistance genes in R. anatipestifer, where the resistance gene pool circulating is not well understood.

**IMPORTANCE** Trimethoprim is a synthetic antimicrobial agent inhibiting dihydrofolate reductase (DHFR), which is encoded by the *folA* gene. Acquired genes that confer trimethoprim resistance due to mutations in the *folA* gene are designated *dfr* and divided into two main families including *dfrA* and *dfrB*. Resistance to trimethoprim is mainly mediated by the acquisition of mobile *dfrA* genes, and most of them were discovered in Enterobacteriales. R. anatipestifer belongs to the Flavobacteriaceae family, and the reservoir of *dfrA* resistance genes in R. anatipestifer has not been fully investigated. A novel trimethoprim resistance gene, *dfrA49*, which was identified and characterized in chromosome and plasmid sequences in this study, increased the MIC of TMP (>256-fold) in E. coli BL21(DE3). Our study expands knowledge about the pool of mobile *dfrA* genes that confer resistance to trimethoprim and broadens the understanding of the host spectrum of *dfrA* family genes.

## INTRODUCTION

Antibiotic resistance is regarded as one of the biggest threats to global health (1). Trimethoprim (TMP) is a synthetic antimicrobial agent that inhibits dihydrofolate reductase (DHFR). DHFR is encoded by the *folA* gene in bacterial chromosomes (2). Trimethoprim resistance can arise due to mutations in the *folA* gene that give rise to trimethoprim-insensitive DHFRs (3). Acquired genes that confer trimethoprim resistance are designated *dfr* and are divided into two main families. The chromosomal *dfrA* genes encode DHFR proteins with 152 to 189 amino acid homologues, designated DfrA, and *dfrB* genes encode the smaller 78 amino-acids DfrB proteins (4). Resistance to trimethoprim is mainly mediated by the acquisition of mobile *dfrA* genes, and most of them were discovered in *Enterobacteriales* (5). Under the new nomenclature rules, some reported *dfrA* variants have been sorted out and renamed (2).

Riemerella anatipestifer belongs to the *Flavobacteriaceae* family and is a Gram-negative, nonmotile, non-spore-forming, rod-shaped bacterium, which causes fibrinous pericarditis, perihepatitis, peritonitis, diarrhea, and neurological symptoms in infected ducks (6). The prevalence of the disease can lead to high mortality rates of ducks and significant economic losses (7). With more extensive studies of the tigecycline resistance gene *tet*(X) (8), more evidence suggests R. anatipestifer as a potential ancestral source of *tet*(X) (9). The potential reservoir of other novel resistance genes in R. anatipestifer was not fully investigated, which is a knowledge gap.

In routine surveillance of antimicrobial resistance of R. anatipestifer, we analyzed the draft genomes of 38 R. anatipestifer isolates and the bacteria carrying *dfrA* variants in the NCBI database. We aimed to characterize a new member of the DfrA family in R. anatipestifer, which is distantly related to any known dihydrofolate reductase, and to explore whether it has the potential to transfer to other pathogens.

## RESULTS

### Phylogenetic analysis of *dfrA49* and functional verification.

**FIG 1:**
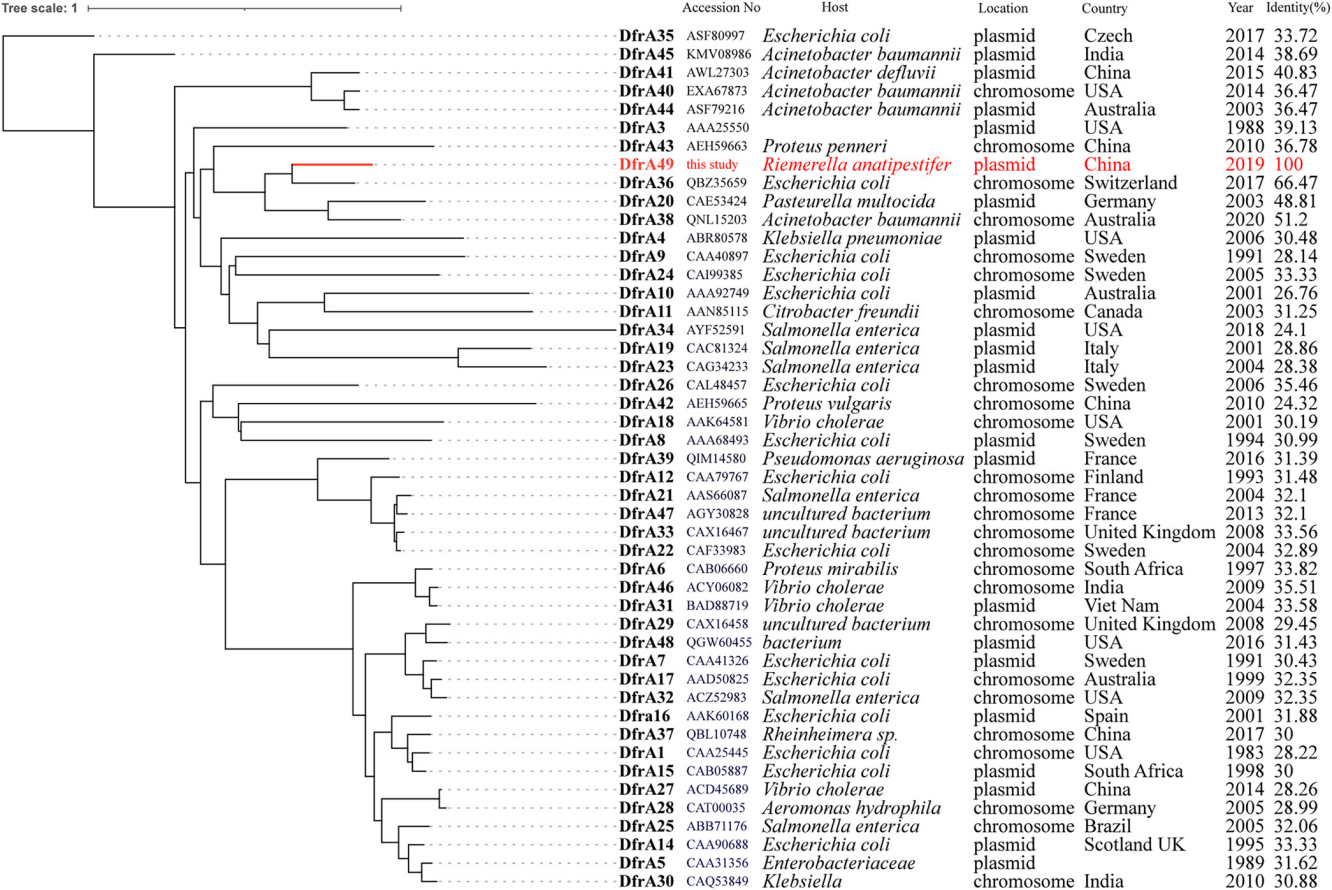
Phylogenetic analysis of amino acid sequences of different *dfrA* variants. Phylogenetic analysis with amino acid sequences of DfrA variants was performed using the neighbor-joining method along with a bootstrap test of 1,000 replicates and the JTT model using MEGA X version 10.0.2. All DfrA protein names for the variants given unique numbers here are in bold while the new DfrA49 is colored in red.

R. anatipestifer isolates were collected from different farms during 2014 to 2020, in Jiangsu and Guangdong provinces in China. In a previous study, the draft genome sequences of 38 R. anatipestifer isolates were obtained successfully by short-read sequencing, and complete genome sequences for 18 of these isolates were generated by combining Nanopore long-read and Illumina short-read sequence. Seven endogenous plasmids were detected in five R. anatipestifer isolates with complete circular sequences (10). To infer an evolutionary relationship among the DfrA family members, we constructed a phylogenetic tree of all available DfrA variants from NCBI database (Fig. 1). The *dfrA49* encoding 167 amino acids was found to share 66.47% identity with DfrA36 and to be located on the same branch with DfrA38 (51.2%) and DfrA20 (48.81%). Other DfrA protein sequences have an amino acid identity ranging from 24.10% to 40.83% to DfrA49.

Among *dfrA49*-positive R. anatipestifer isolates, 100% were resistant to TMP (MIC, >1,024 mg/L). To confirm that the *dfrA49* gene was responsible for the observed trimethoprim resistance, a 504-bp fragment was cloned into pET23a in Escherichia coli BL21(DE3). The resulting transformant was resistant to trimethoprim with an MIC of >1,024 mg/L, relative to BL21(DE3) carrying the empty cloning vector, which had an MIC of ≤2 mg/L (Table 1).

**TABLE 1:** MICs of TMP for *dfrA49*-containing isolates and control strains

Strain/plasmid information	Description	MIC (mg/L) of TMP^*a*^
pET23a	A T7 promoter-driven expression vector without induction	
BL21(DE3)	An expression host of E. coli	
	pET23a-BL21	4 (S)
An E. coli strain harboring a constructed plasmid with *dfrA49*	pET23a::*dfrA49*-BL21	>1,024 (R)
R. anatipestifer reference strain	ATCC 11845	4 (S)
R. anatipestifer isolates without *dfrA49*	20190502E1-H1	≤2 (S)
	20190510E1-1	≤2 (S)
4 R. anatipestifer isolates harboring chromosome-mediated *dfrA49*	20160930RA1	>1,024 (R)
	20190509E1-1	>1,024 (R)
	20190212E1-4	>1,024 (R)
	20190507E1-1	>1,024 (R)
An R. anatipestifer isolate harboring plasmid-mediated *dfrA49*	20190305E2-2	>1,024 (R)

a R, resistant; S, susceptible.

### Genome characterization of *dfrA49*-positive R. anatipestifer isolates.

**FIG 2:**
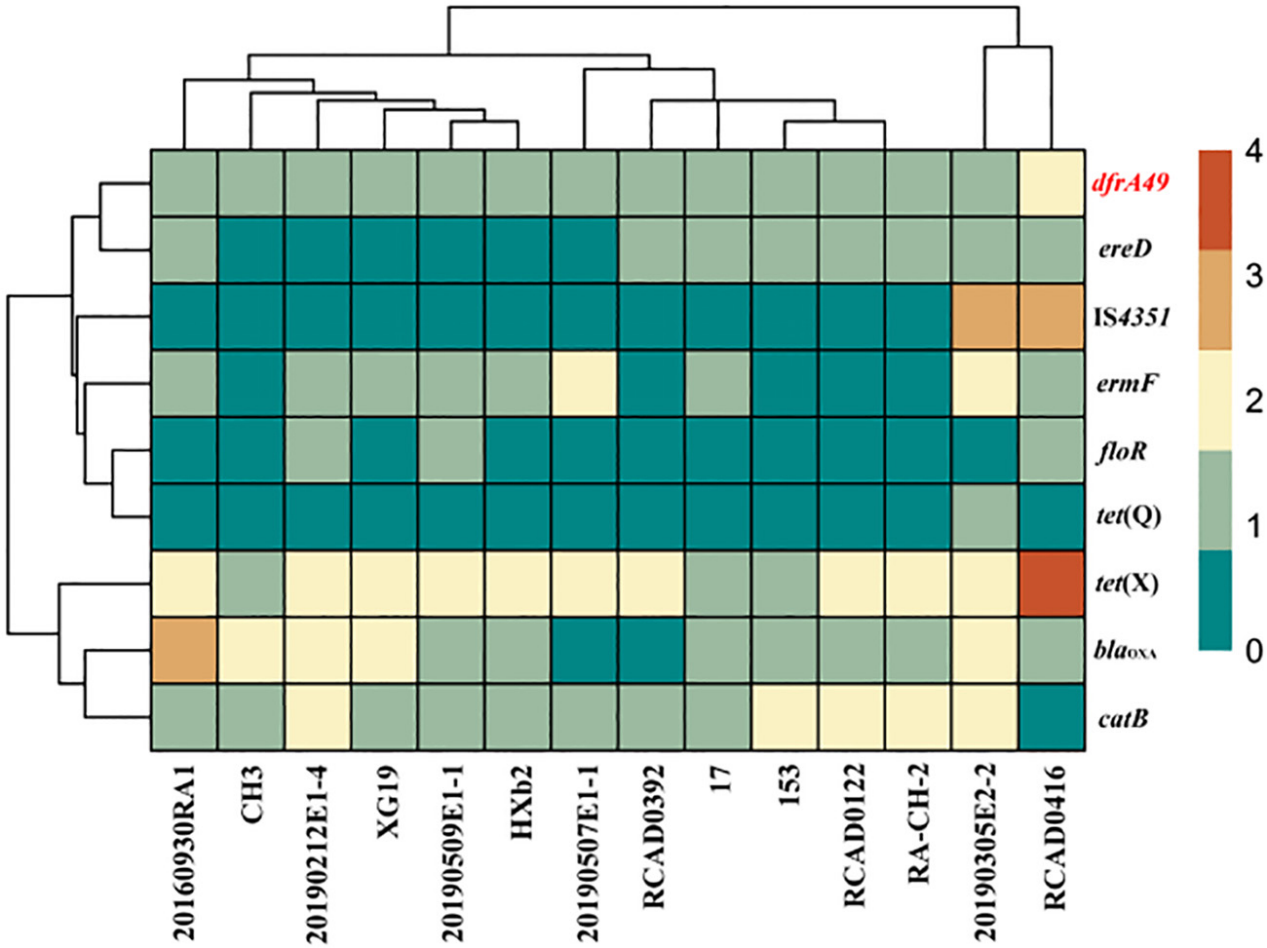
Heat map of resistance genes and insertion sequence distribution in *dfrA49*-positive R. anatipestifer isolates available from the NCBI database. *dfrA49* is colored in red.

**FIG 3:**
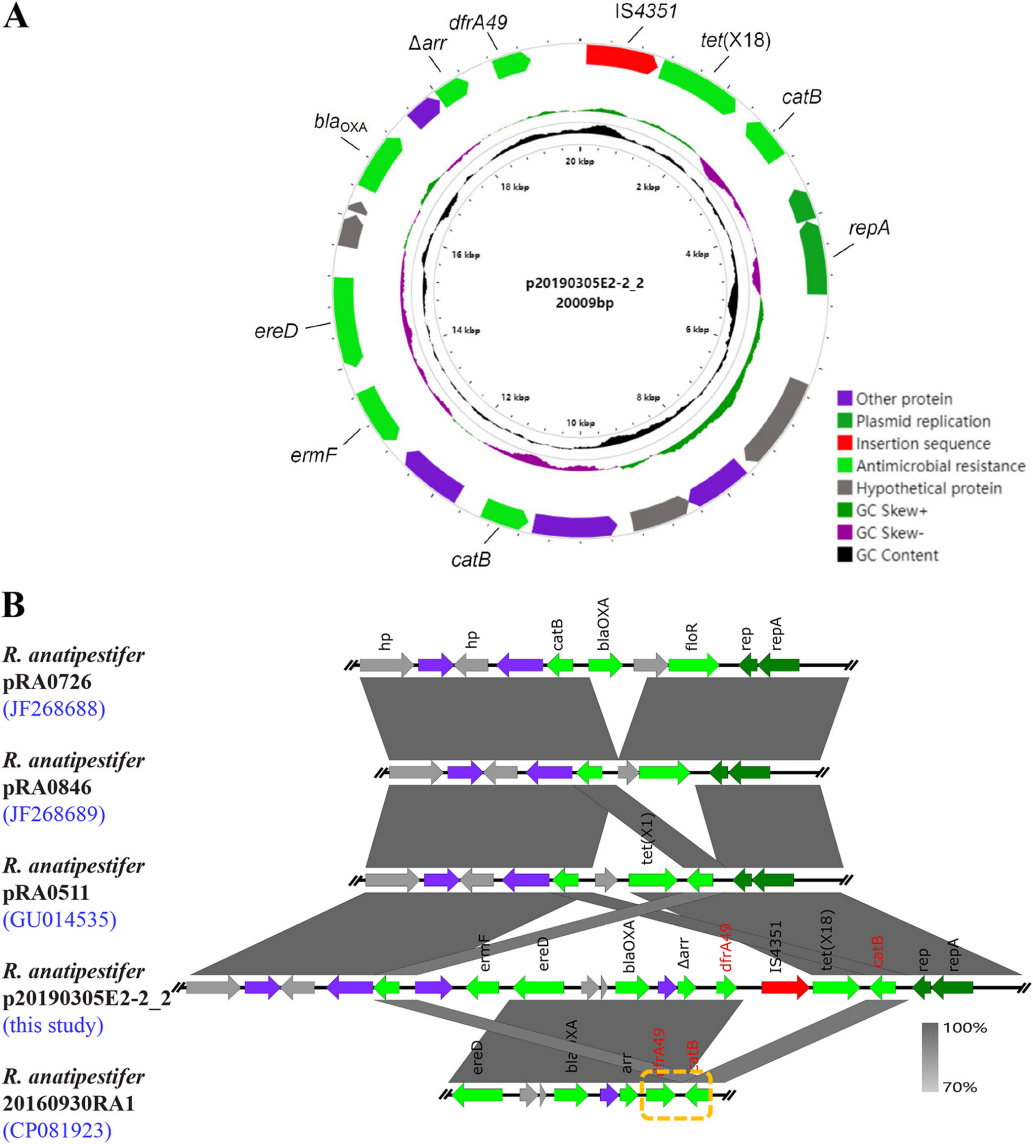
Plasmid profile and genetic environment alignment of p20190305E2-2_2. (A) The plasmid profile of p20190305E2-2_2 from R. anatipestifer isolate 20190305E2-2. Genes with different functions are marked with different colors: purple, other protein; dark green, plasmid replication; red, insertion sequence; light green, antimicrobial resistance gene; gray, hypothetical protein. (B) Comparison of plasmid sequence of p20190305E2-2_2 with other R. anatipestifer plasmids and the genome sequence of R. anatipestifer 20160930RA1. The *dfrA49-catB* structure is marked with a yellow-dashed frame.

Five *dfrA49*-positive isolates were identified in these R. anatipestifer isolates (5/38, 13.16%), and we gained the complete genome sequences. There were abundant antibiotic resistance genes (ARGs) in *dfrA49*-positive isolates, including *catB*, *tet*(X), *floR*, *bla*_OXA_, *ereD*, and *ermF* (Fig. 2). The *dfrA49* gene was found on the chromosomes of four isolates and on one plasmid. We named the plasmid carrying the *dfrA49* gene p20190305E2-2_2 (Fig. 3A). p20190305E2-2_2 (20, 009 bp) has 19 open reading frames (ORFs) and a GC content of 34%, while *dfrA49* has a GC content of 42%. A tentative conjugation assay and a transformation assay failed to demonstrate the transferability of p20190305E2-2_2, indicating its host limitation. Then, we investigated the evolutionary pathway of plasmids harboring the same replicon type with p20190305E2-2_2 by comparing the genetic environments (Fig. 3B). IS*4351* transposase was found around the plasmid-mediated *dfrA49*. Additionally, we screened another 101 R. anatipestifer isolates by PCR and Sanger sequencing from Guangdong Province, China, and found *dfrA49* existed in 24.75% (25/101) of them. Through calculation, we found that the prevalence rate of *dfrA49* in our study was 21.58%.

**FIG 4:**
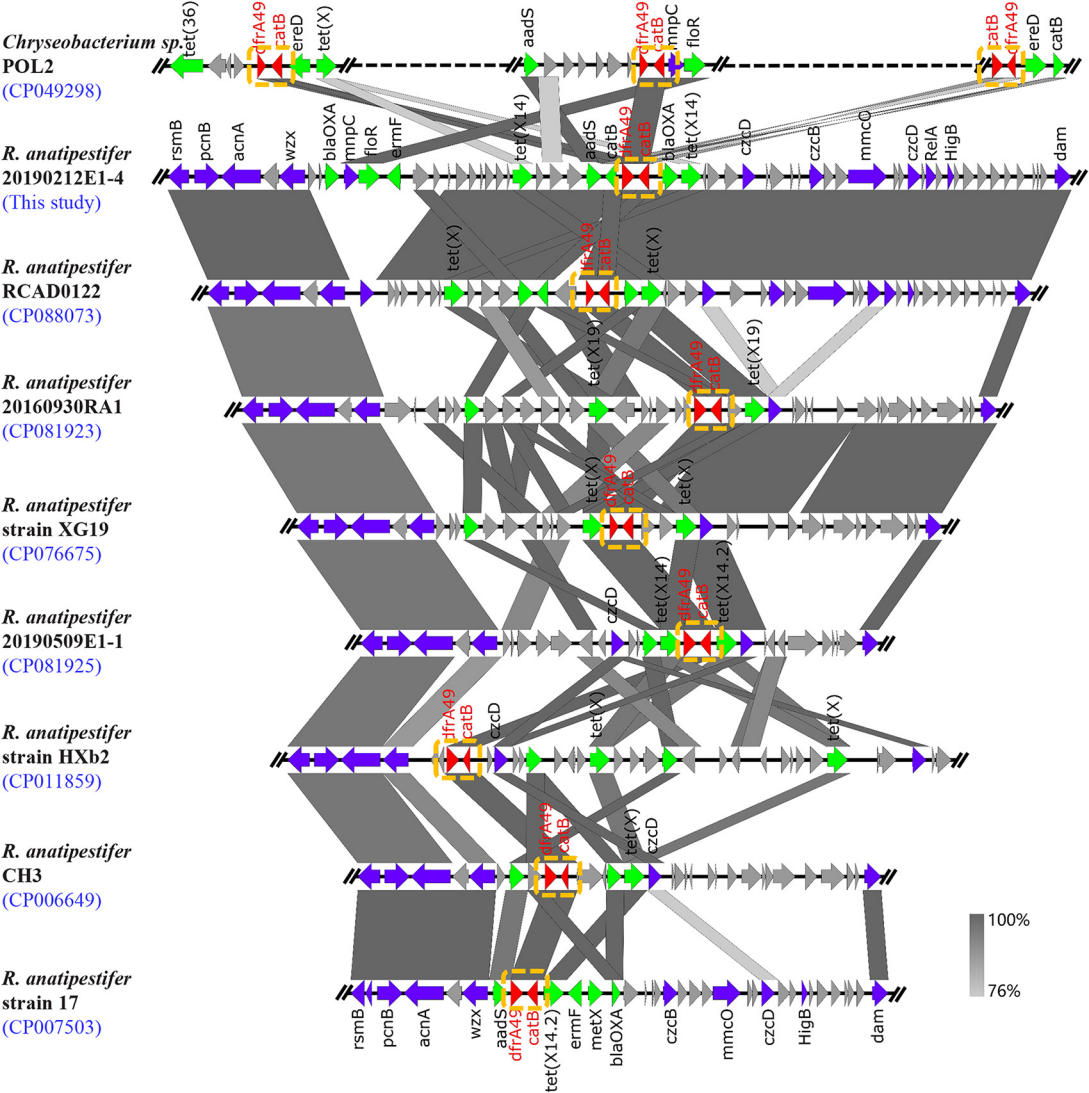
Genetic environments of *dfrA49*-harboring *Flavobacteriaceae* isolates. The arrows denote the direction of transcription of the genes. The functional genes are differentiated by colors. Regions of >76% nucleotide homology are marked by gray shading. The *dfrA49-catB* structure is marked with a yellow-dashed frame.

We screened *dfrA49* according to the whole genome sequencing (WGS) database and nr/nt database from NCBI. A total of 14 R. anatipestifer genomes (five isolates were from our study) and one *Chryseobacterium* sp. genome were found positive for *dfrA49* and downloaded from the NCBI database to analyze the genetic environments. The genetic contexts of dfrA49 were classified to 9 diverse structures and the co-existence of dfrA49 and a catB gene was detected (Fig. 4). In NCBI database, most *dfrA* genes found to date are coupled with a sulfonamide resistance gene. The *catB* gene linked to *dfrA49* in this study belongs to the chloramphenicol *O*-acetyltransferase family. Interestingly, the stable genetic structure *dfrA49-catB* was found not only in R. anatipestifer, but also in *Chryseobacterium* sp., distributed in three noncontiguous regions on the chromosome. The *dfrA49*-*catB* structure was in the multidrug resistance (MDR) region, whether it was located on the chromosome or plasmid.

### Homology modeling of DfrA49.

**FIG 5:**
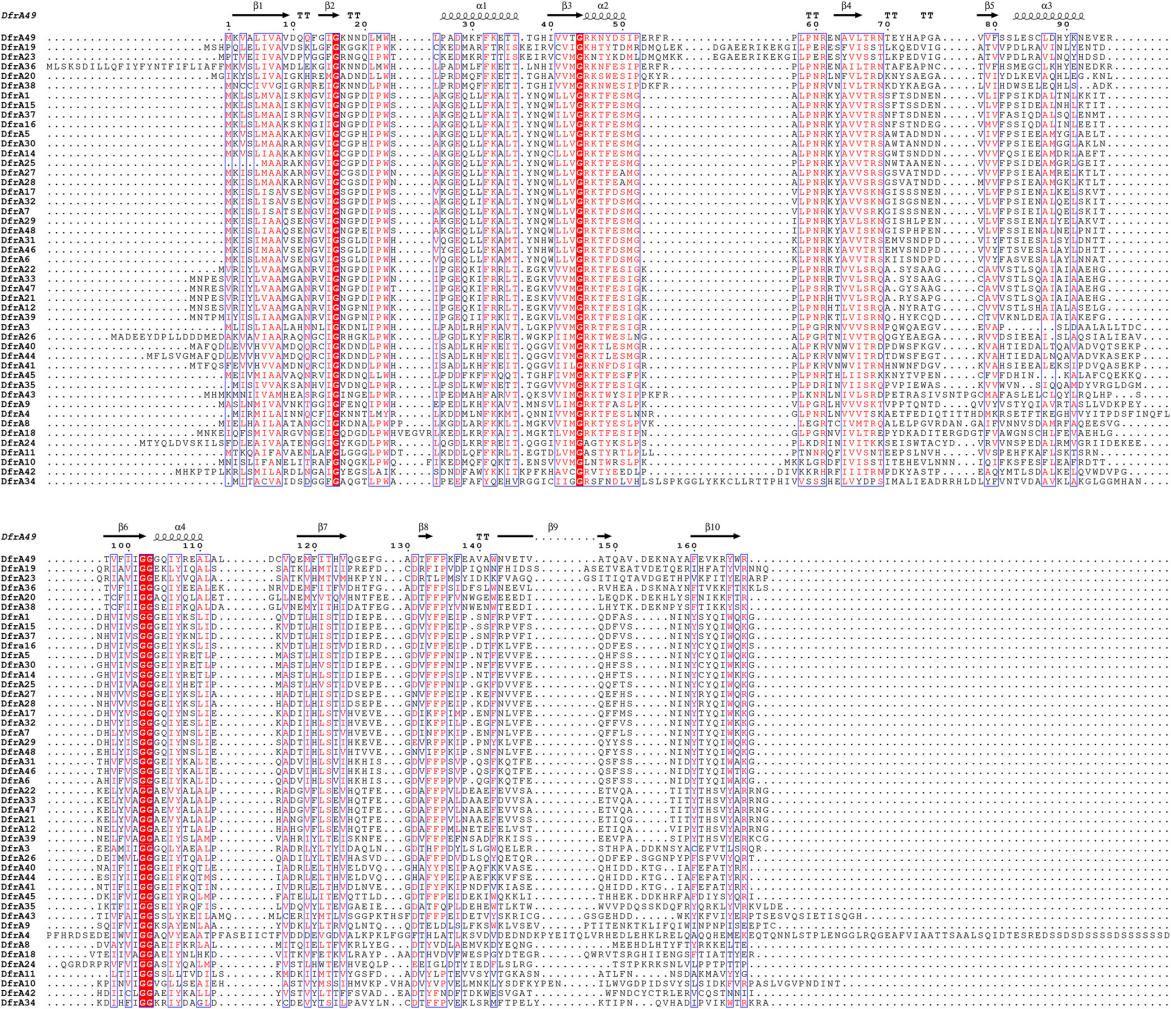
The secondary structure of DfrA49 and its homologues. Multiple sequence alignments were submitted to the CLUSTALW server, and the output was presented with the EndScript server (http://endscript.ibcp.fr/ESPript/cgi-bin/ESPript.cgi). Protein secondary structure is labeled on top. Similar residues are shown as red letters on a white background. Different residues are in a dark font.

**FIG 6:**
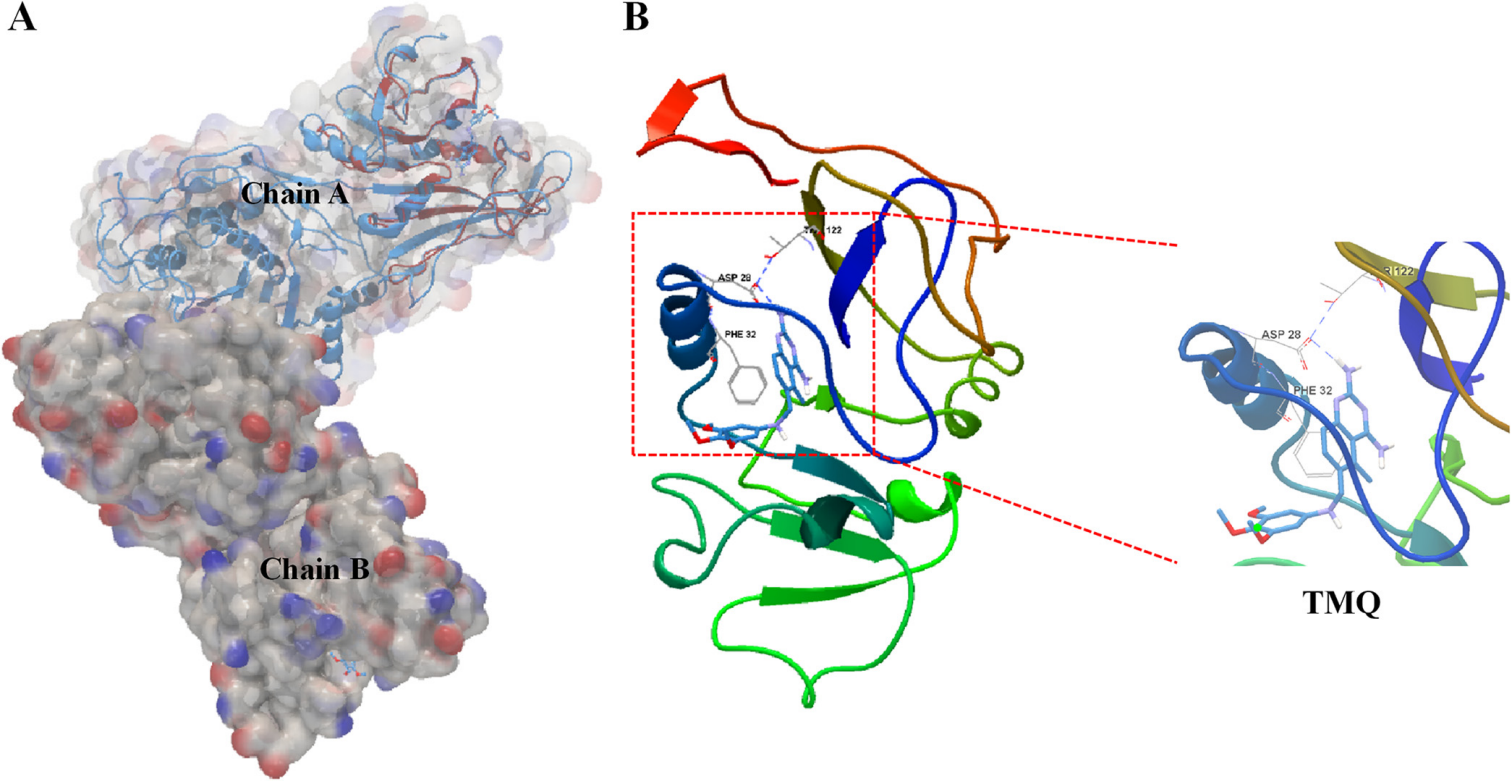
Homology modeling of DfrA49 protein. (A) Superimposed representation of the modeled DfrA49 and template 3hbb.1 structure. (B) The modeling of DfrA49 is based on the published dihydrofolate reductase-thymidylate synthase (template number 3hbb.1) using the online server Swiss-Model (https://swissmodel.expasy.org/) while the sequence identity is 45.86% and global model quality estimate (GMQE) is 0.58. The side chains of residues (Asp28, Phe32, and Thr122) connected with TMQ are indicated in the enlarged views.

The secondary structures of DfrA49 and its homologues were aligned with each other (Fig. 5). The modeling of DfrA49 is based on the published dihydrofolate reductase-thymidylate synthase (template number 3hbb.1), while the sequence identity is 45.86% and the global model quality estimation (GMQE) is 0.58 (Fig. 6B). The model structure of DfrA49 was then superposed onto chain A of the template 3hbb.1 structure to perform a homology modeling analysis (Fig. 6A). Although the homology of amino acids is low, the locations of trimetrexate (TMQ; a nonclassical folic acid inhibitor) small-molecule docking residues were discovered. The side chains of residues connected with TMQ are indicated in the enlarged views in Fig. 6B, including Asp28, Phe32, and Thr122.

## DISCUSSION

Long-term monitoring of ARGs is necessary for epidemiological study of resistance genes and risk evaluation. Surveillance of novel resistance gene variants is important for understanding the evolution and transmission of ARGs. Pathogens resistant to TMP have existed and evolved for a long time, and different *dfrA* genes were discovered successively. In this study, we first reported a new variant, *dfrA49*, encoding dihydrofolate reductase on chromosomes and one plasmid of R. anatipestifer. The deduced DfrA49 protein sequence consists of 167 amino acids and thus is in the size range (152 to 189 amino acids) of most of the bacterial DfrA proteins known so far. As verified by the pET23a expression system using the T7 strong promoter, *dfrA49* in E. coli BL21(DE3) conferred high-level drug resistance to TMP and increased the MIC of TMP (>256-fold) in E. coli. The phenotype of *dfrA49* in E. coli is in accordance with that in R. anatipestifer. Phylogenetic analysis showed that DfrA49 was on the same branch with DfrA20, which was detected on the plasmid pCCK154 (11 kb) from Pasteurella multocida (11) and in clusters containing DfrA proteins from Gram-negative bacteria such as Pseudomonas aeruginosa, E. coli, and Salmonella enterica in the phylogenetic tree (Fig. 1). Identities to the known DfrA proteins based on a multisequence alignment varied between 26.76% and 48.81%. This result confirmed that DfrA49 was distinct from other previously characterized DfrA proteins. Detailed genomic analysis demonstrated that *drfA49* always coexisted with the *catB* gene in R. anatipestifer isolates. According to a previous study, most *dfrA* genes were found associated with IS*CR1* located downstream of *sul1* in a complex class 1 integron, or associated with IS*CR2*, which is frequently surrounded by the *sul2* gene (2). To the best of our knowledge, *dfrA49* associated with variable regions embedded downstream of the *catB* gene revealed a new genetic structure of the *dfrA* genes. *Chryseobacterium* sp. strain POL2 containing copies of chromosome-mediated *dfrA49* in three noncontiguous variable regions also harbored the structure *dfrA49*-*catB* separately. The structure was found constantly surrounded by two conservative chromosomal loci in R. anatipestifer, and a total of nine different genetic contexts of *dfrA49-catB* structure were obtained by linear alignment (Fig. 4). The *dfrA49-catB* structure was in MDR regions carrying *tet*(X) variants between *wzx* and *czcD*. Coincidentally, both R. anatipestifer and *Chryseobacterium* sp. belong to the family *Flavobacteriaceae*. Whether the *dfrA49-catB* structure only occasionally exists or is highly prevalent in *Flavobacteriaceae* strains needs continuous follow-up monitoring.

Since the plasmid pCFC1 (NC_002111) in R. anatipestifer was first reported by C. F. Chang in 1997 (12), pCFC2 (NC_002130), JX (KY806579), pRA0726 (NC_015956), pRA0846 (NC_015950), pRA0511 (NC_019261), and pRCAD0416RA-1 (CP073240) have been identified in R. anatipestifer continually. Our previous study revealed seven endogenous plasmids, containing only one MDR plasmid, p20190305E2-2_2, carrying *dfrA49*, *catB*, *ermF*, *ereD*, *bla*_OXA_, Δ*arr*, and *tet*(X18). In addition, the *bla*_OXA_ variant share 88.36% identity with *bla*_OXA-209_; a study of resistance genes of this family will be further performed. It was worth noting that the *dfrA49*-*catB* structure in plasmid p20190305E2-2_2 was possibly interrupted by the insertion of IS*4351*-*tet*(X18) (Fig. 3B). This phenomenon indicated that there might be hot spot sites of insertion between *dfrA49* and *catB*. Bacteria carrying the *dfrA49-catB* structure are at risk of ARG transfer and enrichment based on potential transposable hot spots.

The revolution in genome sequencing has provided a wealth of information that continues to reveal novel complete genome maps, but manual curation and experimental evidence are needed to investigate the functions of genes (13). In the results from sequencing assembly, the distinction between plasmids and chromosome fragments needs to be confirmed with reference to the original reads. The identification and analysis of plasmids in R. anatipestifer might need to be more rigorous, with the replicon as an important reference. By comparing the genetic environments of plasmids, we investigated the evolution of plasmids harboring the same replicon in R. anatipestifer (Fig. 3B). We speculated that the plasmid p20190305E2-2_2 might have acquired MDR regions from the chromosome of R. anatipestifer 20160930RA1. The plasmid may further exchange ARGs with other plasmids and chromosomes, resulting in the enrichment and spread of ARGs in different clones.

In conclusion, a novel *dfrA49* conferring TMP resistance was identified from a new genetic environment from R. anatipestifer. The emergence of *dfrA49* heralds the importance of R. anatipestifer as the ARG reservoir. As a potential ancestral source of *tet*(X), R. anatipestifer has attracted much attention from researchers recently. R. anatipestifer plays a more important role in the field of public health and food safety. The transmission ability across bacterial species boundaries and the epidemiology of *dfrA49* and other potential resistance genes in this bacterial species warrant continuous surveillance and investigations.

## MATERIALS AND METHODS

### Bacteria, growth conditions, and detection of *dfrA49*.

R. anatipestifer isolates were obtained from a previous surveillance study (10). The species of the isolates were identified by matrix-assisted laser desorption ionization time of flight mass spectrometry MALDI-TOF MS analysis (14). R. anatipestifer isolates were cultured at 37°C in tryptic soybean broth (TSB) or tryptic soybean agar (TSA) plates with 5% calf serum (15). E. coli BL21(DE3) was grown at 37°C in Luria-Bertani (LB) broth or on LB agar plates. The presence of the *dfrA49* gene was checked using PCR with specific primers dfrA49-P1 (CCCCGCATGGTGTTCATTTC) and dfrA49-P2 (GCTTGGGTTGCTACCGTTTC) designed in this study. All PCR amplicons were sequenced and underwent BLASTn to confirm the correct amplifications.

### WGS and bioinformatics analysis.

To obtain genetic information for R. anatipestifer isolates, high-quality genomic and plasmid DNA was extracted with the FastPure bacterial DNA isolation minikit (Vazyme, China) in accordance with the manufacturer’s recommendation. Whole-genome sequencing was performed via the Illumina HiSeq 2500 platform and the Oxford Nanopore Technologies MinION platform to acquire the short-read and long-read data. Short-read and long-read data were combined to generate complete genome sequences using a hybrid assembly strategy (16). Antibiotic resistance genes (ARGs) were identified using ResFinder 3.2 (17). The heat map was drawn using the pheatmap R package. A total of 48 amino acid sequences of DfrA were searched and downloaded according to the new *dfrA* naming guideline from the NCBI database. Phylogenetic analysis with amino acid sequences of DfrA variants was performed using the neighbor-joining method along with a bootstrap test of 1,000 replicates and the JTT model using MEGA X version 10.0.2 (18). The phylogenetic tree was visualized by iTOL v6 (https://itol.embl.de/itol.cgi) (19). To perform alignments, the amino acid sequences of DfrA variants were uploaded to the CLUSTALW server (https://www.genome.jp/tools-bin/clustalw). Then, the output alignment sequence was submitted to the ESPript 3 server (http://endscript.ibcp.fr/ESPript/ENDscript/) to predict secondary structures.

### Transformation assay of the *dfrA49* gene.

Conjugation experiments and horizontal transfer of p20190305E2-2_2 via electroporation were performed to explore the resistance level of chromosome- and plasmid-mediated *dfrA49*. The plasmid p20190305E2-2_2 was extracted with the EndoFree Maxi plasmid kit (Tiangen Biotech Co., Ltd., Beijing, China) and checked by the ND-2000. Different competent cells, including E. coli DH5α, E. coli S17-1, E. coli C600, and R. anatipestifer ATCC 11845, were tested as recipient strains separately. Transformants were selected using LB agar plates containing trimethoprim (100 mg/L) after incubation at 37°C for 24 h.

### Functional analysis of the *dfrA49* gene in E. coli.

To verify the activity of *dfrA49*, the intact ORF of *dfrA49* was amplified by PCR with primers pET23a-dfrA49-F (c***gagctc***GCCAGGGGAACACAGGTTCA) and pET23a-dfrA49-R (ccc***aagctt***CGACAGCTTCGAATTTCGGG) and enzyme cleavage sites were indicated by boldface italic underline. The amplified products and expression vector pET23a (T7 promoter) were digested with SacI and HindIII, subjected to ligation, and transformed into E. coli BL21(DE3) competent cells by heat shock (20). The successful transformants were grown overnight at 37°C in LB broth containing 100 mg/L ampicillin under continuous shaking and confirmed by Sanger sequencing.

### Antimicrobial susceptibility testing.

The MICs of TMP for E. coli BL21(DE3) with the pET23a expression system were determined using the broth microdilution methods in accordance with the Clinical and Laboratory Standards Institute (CLSI) guidelines (19). E. coli BL21(DE3) with the pET23a vector without the DNA insert was used as a control. R. anatipestifer isolates were cultured for antimicrobial susceptibility testing against the two antibiotics based on our previous study (10). R. anatipestifer ATCC 11845 and E. coli ATCC 25922 were used as controls.

### Structural modeling of DfrA49.

The amino acid sequences of DfrA49 were submitted to the online server Swiss-Model (https://swissmodel.expasy.org/) to construct three-dimensional (3D) structures. 3hbb (PDB entry code) was employed as the template. The overlaps of structures of DfrA49 and the template 3hbb were generated to compare spatial conformational differences in CLC Genomics Workbench.

### Ethical approval.

Ethical approval was not required.

### Data availability.

The plasmid p20190305E2-2_2 harboring *dfrA49* was deposited in the NCBI database with accession number CP072192. Chromosomes of five *dfrA49*-positive isolates were submitted in the NCBI database with accession numbers CP081925 (20190509E1-1), CP081923 (20160930RA1), CP081934 (20190507E1-1), CP081924 (20190212E1-4), and CP072190 (20190305E2-2).
